# Pediatric TSH Reference Intervals and Prevalence of High Thyroid Antibodies in the Lebanese Population

**DOI:** 10.1155/2017/6372964

**Published:** 2017-01-18

**Authors:** Marie-Hélène Gannagé-Yared, Nicole Balech, Vanessa Farah, Marianne Antar, Rindala Saliba, Elise Chahine

**Affiliations:** ^1^Department of Endocrinology, Faculty of Medicine, Saint Joseph University, Beirut, Lebanon; ^2^Hormonology Laboratory, Hôtel-Dieu de France Hospital, Beirut, Lebanon

## Abstract

The aims of this study are to establish reference values for TSH in Lebanese schoolchildren; to look at the relationship between TSH and age, gender, BMI, socioeconomic status (SES), and thyroid antibodies (TAb); and to investigate the prevalence of abnormal TAb in this population. 974 Lebanese schoolchildren aged 8–18 years were recruited from 10 schools of different SES. Third-generation TSH, TPO-Ab, and Tg-Ab measurements were performed using the IMMULITE chemiluminescent immunoassay. The mean TSH is 2.06 ± 1.05 *μ*UI/ml. TSH values are inversely correlated with age (*p* < 0.0001), are higher in boys than in girls (resp., 2.14 ± 1.10 and 1.98 ± 0.99 *μ*UI/ml, *p* = 0.017), and are positively correlated with BMI (*p* < 0.0001). They are also significantly higher in subjects from low-SES schools (*p* = 0.03) and in girls with positive TAb (*p* = 0.026). In boys, TSH is independently associated with age, BMI, and schools' SES (*p* = 0.01, *p* = 0.03, and *p* = 0.026, resp.) while in girls, the association is only significant for age and TAb (*p* = 0.0001 and *p* = 0.015, resp.). The prevalence of TAb is 4.3% (3% for TPO-Ab and 2.1% for Tg-Ab). Our results showed higher TSH values in the pediatric Lebanese population compared to western populations. TSH varies according to age, gender, BMI, and SES and is associated in girls with TAb.

## 1. Introduction

Thyroid dysfunction is one of the most commonly encountered endocrine disorders in clinical practice. The American Thyroid Association (ATA) considers TSH measurement as the best useful test for the thyroid function evaluation [1]. Therefore, establishing a reference interval in a population allows clinicians to assess thyroid function and reduce patient classification errors [2].

TSH and its relationship with several parameters including age, sex, ethnicity, obesity, and thyroid antibodies have been the subject of numerous studies [3–11]. TSH decreases with age in the pediatric population [4, 9, 10]. It also varies with sex, with discrepant results between studies. In the adult population, the United States (US) National Health and Nutrition Examination Survey (NHANES III) [5] shows significant higher values in women compared to men; conversely, in the Boucai et al. study [12], women have lower 2.5th and 50th TSH percentiles than men, but gender does not affect the 97.5th percentile. In the pediatric population, in two studies, no significant differences in TSH values were reported between boys and girls [9, 11], while in a third one, the TSH decline with age was faster for females [10]. Finally, higher TSH values were observed in obese children compared to normal-weight ones [6, 7].

Hashimoto's thyroiditis is known to be the most common form of thyroiditis in infancy. García-García et al. [13] showed that 3.7% of Spanish children and adolescents have positive thyroid antibodies (2.3% for thyroid peroxidase antibodies (TPO-Ab) and 3% for thyroglobulin antibodies (Tg-Ab)). On the other hand, in the NHANES III study, the prevalence of positive Tg-Ab and TPO-Ab in adolescents is higher with respective values of 6.3% and 4.8% [5]. Furthermore, Boucai et al. [12] showed, in a US population aged 13 to more than 80 years, that the presence of thyroid antibodies increases the 97.5th percentile of TSH values, while, at the opposite, in children and adolescents, another study showed minimal influence of thyroid antibodies on TSH reference range [8].

The TSH reference intervals may vary from one population to another. The relationship between TSH and age, thyroid antibodies, gender, body mass index (BMI), socioeconomic status (SES), and the prevalence of thyroid antibodies (TPO-Ab and Tg-Ab) has never been studied in a Middle Eastern population.

The aims of our study are (1) to establish reference values for TSH in the pediatric Lebanese population; (2) to evaluate the relationship between TSH and age, gender, BMI, socioeconomic status (SES), and thyroid antibodies; and (3) to assess the prevalence of thyroid antibodies in our population.

## 2. Subjects and Methods

### 2.1. Population

In this cross-sectional study, ten private and public schools were targeted for recruitment using a randomized stratified sampling. These schools were selected from the areas of the Great Beirut and Mount Lebanon, both areas concentrating the majority of the Lebanese population. Recruitment was done between May 2013 and October 2014. Schools were categorized as high, middle, or low SES depending on the yearly school fees (resp., for group 1 between $5000 and $7000; for group 2 between $3000 and $5000; and for group 3 between $1500 and $3000 or free, the last group corresponding to public or semipublic schools). Children with known thyroid disorders or with any chronic medical condition (such as diabetes and renal, hepatic, cardiac, or pulmonary diseases) or taking medications that might have affected TSH values such as corticosteroids were excluded from the study, as well. None of the subjects presented with genetic syndromes or chromosomal abnormalities. One should mention that all Lebanese newborns are screened for congenital hypothyroidism, and none of the included subjects were known to have congenital hypothyroidism. A written informed consent was signed by the children's parents. Information regarding previous medical history or concomitant medical treatment was provided by the parents via a questionnaire. The protocol was approved by our university ethics committee (CEHDF449).

Nonfasting sampling was performed on all subjects in the schools between 8 and 10 am. The day of sampling, height, and weight were measured for all participants using the same device. BMI was calculated as weight in kilograms divided by height in meters squared (kg/m^2^). To account for variability by age and sex, all BMI measures were compared with age- and sex-specific reference values, from the 2000 CDC growth charts in order to define the weight status [14]. Normal weight was defined as a BMI-for-age-sex of <85th percentile, overweight as a BMI-for-age-sex of the 85th to <95th percentile, and obesity as a BMI-for-age-sex of ≥95th percentile [14]. The population was categorized into three age groups: 8–11 years, 12–14 years, and 15–18 years.

### 2.2. Laboratory Analysis

Blood specimens were then centrifuged the day of sampling, and the serum was subsequently frozen at −80Â°C. TSH was measured by a solid-phase, two-site chemiluminescent immunometric assay (IMMULITE 2000 Third-Generation TSH, Siemens Corp., Tarrytown, NY). Thyroid peroxidase autoantibodies (TPO-Ab) as well as thyroglobulin autoantibodies (Tg-Ab) concentrations were determined with enzyme-labeled, chemiluminescent sequential immunometric assays (Siemens, IMMULITE 2000 Anti-TPO-Ab, IMMULITE 2000 Anti-Tg-Ab, USA). For TSH, the analytic sensitivity of the kit is 0.004 *μ*UI/ml, and the coefficient of variation is less than 5.5% for TSH values comprising between 0.3 and 10 *μ*UI/ml. For TPO-Ab and Tg-Ab, respective analytic sensitivities are 5 UI/ml and 2.2 IU/ml, while, for both parameters, intra-assay coefficients of variation are <7%. The TSH reference interval provided by the manufacturer in subjects aged 13 to 20 comprised between 0.4 and 4 *μ*UI/ml while for the age group of 2 to 12 years, it is 0.58–4.1 *μ*UI/ml. The cutoff points for anti-Tg-Ab and anti-TPO-Ab are 40 IU/ml and 35 IU/ml, respectively.

### 2.3. Statistical Analysis

Data were analyzed using SPSS Version 21. Results were expressed as mean ± standard deviation. Comparison of continuous variables was performed using a 2-tailed *t*‐test. Linear correlations, using the Pearson correlation coefficient, were performed to study the correlation between variables. ANOVA tests were used to compare means between groups. Chi-square independence tests were used to search for the association between two categorical variables. Finally, multilinear regression analyses were used to determine the independent predictors of TSH levels.

## 3. Results

### 3.1. Anthropometric and Socioeconomic Characteristics of the Population

The population includes 974 subjects (508 boys (52.2%) and 466 girls (47.8%)). Baseline characteristics of the population are shown in Table 1. There is no significant difference in age between boys and girls (*p* = 0.8). Boys have higher BMI than girls, although this difference is not statistically significant (*p* = 0.06). The distribution of the population according to age groups, BMI categories, and schools' SES is shown in Table 2.

### 3.2. TSH Values according to Sex, Age, and BMI

**Figure 1:**
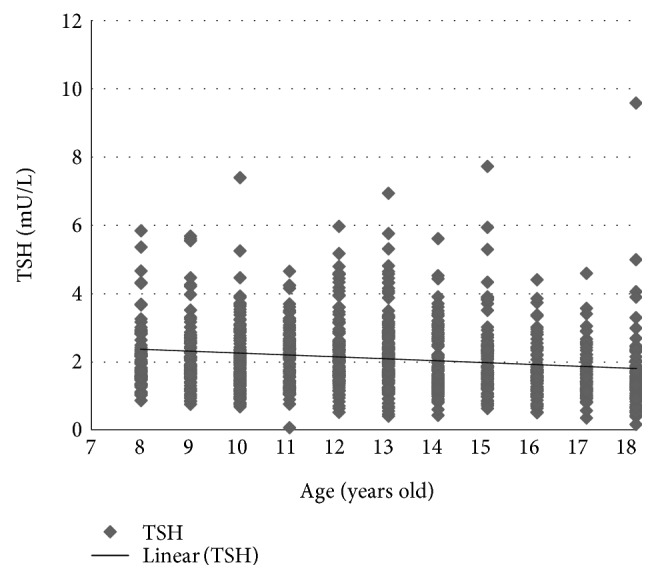
Distribution of TSH according to age categories.

The mean TSH value is 2.06 ± 1.05 *μ*UI/ml in the whole population. TSH distribution according to age categories in the total population and in boys and girls separately is shown in Table 3 and Figure 1. There are significantly higher TSH values in boys compared to girls (resp., 2.14 ± 1.10 *μ*UI/ml and 1.98 ± 0.99 *μ*UI/ml, *p* = 0.017). TSH is inversely correlated with age in the overall population, as well as in boys and in girls separately ( *r* = −0.15, *p* < 0.0001; *r* = −0.14, *p* = 0.002; and *r* = −0.18, *p* < 0.0001, resp.).

TSH varies according to BMI (mean values 2.59 ± 1.43 *μ*UI/ml in obese subjects, 2.11 ± 0.98 *μ*UI/ml in overweight subjects, and 1.95 ± 0.94 *μ*UI/ml in normal-weight subjects). It is positively correlated with BMI in the overall population ( *r* = 0.124, *p* < 0.0001), as well as in boys and girls separately (in boys *r* = 0.136, *p* = 0.002; in girls *r* = 0.1, *p* = 0.03).

TSH was then analyzed according to age categories. It was higher in boys compared to that in girls in both the age groups 8–11 and 15–18 ( *p* = 0.014 and *p* = 0.005, resp.). This difference persisted for both groups after adjustment for BMI ( *p* = 0.011 and *p* = 0.022, resp.).

### 3.3. TSH Values according to Schools' SES

TSH varies according to schools' SES. Respective TSH values in high-, middle-, and low-SES schools are 1.97 ± 0.98 *μ*UI/ml, 2.00 ± 1.06 *μ*UI/ml, and 2.16 ± 1.07 *μ*UI/ml, with a significant difference between the three groups (*p* = 0.03).

### 3.4. Prevalence of Abnormal TSH

In the overall studied population, 53 patients (5.4%) show a TSH ≥4 *μ*UI/ml, 5.7% in boys and 5.2% in girls with no significant gender difference (*p* = 0.7). None of the studied children have a TSH level higher than 10 IU/ml, whereas only two show a TSH value below 0.3 *μ*UI/ml, one of whom was diagnosed with subclinical Graves' disease.

### 3.5. Prevalence of Abnormal Thyroid Antibodies and Their Relation with TSH, Age, and Sex

Forty-two subjects (4.3%) (15 boys (2.9%) and 27 girls (5.8%)) have positive thyroid antibodies (TPO-Ab and/or Tg-Ab), this gender difference being significant (*p* = 0.029). Twenty-nine subjects (3%) have positive TPO-Ab and 20 (2.1%) positive Tg-Ab. In subjects with positive thyroid antibodies, the respective mean TPO-Ab and Tg-Ab values are 215 ± 335 U/l and 333 ± 657 U/l. TSH values are significantly higher in the 27 girls with positive thyroid antibodies compared to those with negative ones (resp., 2.39 ± 1.22 *μ*UI/ml versus 1.96 ± 0.97 *μ*UI/ml, *p* = 0.026). However, this difference is nonsignificant in the overall population and in boys ( *p* = 0.48 and *p* = 0.21, resp.). In the 3 age groups 8–11 years, 12–14 years, and 15–18 years old, respectively, 12, 14, and 16 subjects (i.e., 3.6%, 4.1%, and 5.4%) have positive thyroid antibodies with no statistically significant difference between the 3 groups (*p* = 0.53).

### 3.6. Multilinear Regression Analysis

A multivariate regression analysis was performed in order to study the independent variables that affect TSH values. Variables that were found to be significantly associated with TSH in the bivariate analysis were entered in the regression. In the overall population, TSH was found to be independently associated with sex, age, BMI, and schools' SES ( *p* = 0.018, *p* < 0.0001, *p* = 0.001, and *p* = 0.008, resp.), but not with thyroid antibodies. In boys, this independent association was found with age, BMI, and schools' SES ( *p* = 0.001, *p* = 0.003, and *p* = 0.026, resp.), while in girls, it was only significant for age and thyroid antibodies ( *p* < 0.0001 and *p* = 0.015, resp.) (Table 4).

## 4. Discussion

Our study aimed to establish reference values for TSH in a sample of the Lebanese pediatric population. We observed that the median TSH value was higher in our population compared to the values established by the manufacturer in the US population (resp., 1.98 *μ*UI/ml versus 1.53–1.56 *μ*UI/ml in the age group 8–11 years). In addition, in two NHANES III reports [3, 12], the median TSH in the US population for the ages 12–19 showed lower values than those of ours (resp., 1.37 and 1.30 *μ*UI/ml). Furthermore, we found that 5.4% of our subjects have TSH values ≥4 *μ*UI/ml, while only 2.7% of US adolescents aged 12–19 have TSH values ≥4.5 *μ*UI/ml [3]. The higher cutoff used in this last study may explain part of this difference. Lower iodine status in Lebanese children [15] or genetic/ethnic factors could also be behind this, since both factors were found to affect the TSH reference interval [16–20].

We then studied the relationship between TSH and age, sex, BMI, and schools' SES. We found that TSH decreases with age, which is in line with several other studies [4, 9, 10]. Possible explanations for this finding include a change in negative feedback relationships between free T4 and TSH or changes in TSH bioactivity. In addition, we observed significant higher TSH values in boys compared to girls, a relation that was independent of BMI, suggesting that sex steroids may play a role in TSH regulation. In the literature, the relation between gender and TSH shows controversial results. Three studies [4, 9, 11] did not find significant differences in TSH according to sex, while, in a fourth study, and in line with our results, a faster decline of TSH with age was observed in females [10]. The reason behind these differences is unknown. Possible explanations might be a larger age range (1 day to 18 years) [9, 11], a hospital-based recruitment [9], a smaller sample size population [11], higher BMI in our male population, or ethnic differences.

As reported in other studies [6, 7, 21, 22], TSH was positively correlated with BMI. Several mechanisms leading to higher TSH values in obesity have been suggested. Leptin concentrations may partly explain obesity's effects on thyroid status, perhaps through leptin's effects on TSH secretion [23, 24]. Derangement in the hypothalamic‐pituitary axis, impaired feedback due to lower number of T3 receptors in the hypothalamus, and a decrease in peripheral deiodinase activity have been evoked too [25]. We found that the relationship between TSH and BMI is significant in both sexes but is only independent of other covariates in boys. While two previous studies showed higher TSH values in obese children/adolescent populations [6, 7], in a third study [8], BMI had no influence. The reason behind the lack of an independent effect of BMI in our female population is unknown and could be the lower weight of our girls compared to boys.

To our best knowledge, no previous studies evaluated the relationship between TSH and SES level. We found a significant difference in TSH values between the 3 different schools' SES, with higher values in children from low-SES schools. This finding may be due to the lack of seafood consumption and subsequently to lower iodine intake in children from lower-SES schools, since seafood is an important iodine source in Lebanon.

Finally, we found that the prevalence of positive thyroid antibodies in our population is 4.3% (3% for TPO-Ab and 2.1% for Tg-Ab). This prevalence is similar to the one observed in the Spanish population [13] (which is 3.7%), but lower than the ones reported in the adolescent population of the NHANES III study [5] (which is, resp., 6.3% and 4.8% for Tg-Ab and TPO-Ab). This lower prevalence in our study compared to that in the US population could be related to an insufficient iodine intake [15], since iodine excess is a known risk factor for autoimmune thyroid disease [26, 27]. In addition, we found that the presence of thyroid antibodies is a determinant of TSH values in girls but not in boys. While the results of Kratzsch et al. showed no effect of thyroid antibodies on TSH reference intervals during the first decades of life [8], Boucai et al. [12] found in a population aged 13 to over 80 that the 97.5th TSH percentile was affected by these antibodies. Moreover, a more recent study showed that higher TSH in healthy children was not associated with a higher prevalence of TPO-Ab [20], confirming the results we found in boys. On the other hand, we did not find an association between age and positive thyroid antibodies, even though a tendency towards a nonsignificant higher prevalence of positive thyroid antibodies in our adolescent population was noted. Conversely, two studies showed that the prevalence of positive TPO-Ab is conditioned by age [28] and increases during puberty [29] in childhood and adolescents' populations. This discrepancy with our study could be related to the small number of positive thyroid antibodies in our population. Finally, we found a gender difference in the prevalence of thyroid antibodies with a male to female ratio of 1/2. This finding could be explained by sex steroids or skewed X-chromosome inactivation. Similarly, chronic autoimmune thyroiditis in children and adolescents has shown a lower male to female ratio compared to adult autoimmune thyroiditis (1/4.2 versus 1/10) [30].

Our study may present some limitations. First, because of its cross-sectional nature, it does not look at individual changes over time. Second, the iodine status of our population is lacking. Finally, the normal limits for serum TSH vary greatly depending on the used method [31].

In conclusion, our study showed that the median TSH values are higher in our population compared to the US one, while the prevalence of positive thyroid antibodies is lower. To explain these differences, there is a need to assess the relationship between iodine status and both thyroid function and antibodies. Moreover, it would be interesting to follow over time subjects with abnormal TSH, since subclinical hypothyroidism in children seems to be a remitting process with a low risk (ranging from 0 to 28%) of evolution toward overt hypothyroidism [32, 33]. Finally, because recent studies show in children an association between subclinical hypothyroidism and cardiovascular risk factors [34], an association that is improved after L-thyroxine therapy [35], further studies are needed in our population to assess the long-term effect of higher TSH levels on cardiovascular risk factors.

## Figures and Tables

**Table 1:** Anthropometric characteristics of the population. Data are expressed as mean ± SD.

	Total population *n* = 974	Boys *n* = 508	Girls *n* = 466
Age (yrs.)	13.37 ± 2.93	13.39 ± 2.82	13.35 ± 3.04
Weight (kg)^*∗*^	52.48 ± 17.28	55.60 ± 19.54	49.07 ± 13.66
Height (cm)^*∗*^	156.36 ± 14.59	159.26 ± 16.06	153.21 ± 12.05
BMI (percentiles)	61.50 ± 29.71	63.22 ± 30.36	59.63 ± 28.89

^*∗*^ *p* < 0.0001 between boys and girls.

**Table 2:** Distribution of the population according to age groups, BMI categories, and SES.

		Total population		Boys		Girls	
		*N*	%	*n*	%	*n*	%
Age	8–11	333	34.2%	159	31.3%	174	37.3%
	12–14	344	35.3%	198	39.0%	146	31.3%
	15–18	297	30.5%	151	29.7%	146	31.3%

BMI percentiles	≥95	123	12.6%	81	15.9%	42	9.0%
	≥85 and <95	175	18%	102	20.1%	73	15.7%
	≥5 and <85	646	66.3%	312	61.4%	334	71.7%
	<5	30	3.1%	13	2.6%	17	3.6%

Schools' SES	High	253	26.0%	137	27.0%	116	24.9%
	Middle	258	26.5%	127	25.0%	131	28.1%
	Low	463	47.5%	244	48.0%	219	47.0%

**Table 3:** TSH values according to age and sex in the overall population.

		TSH					
		Mean	Median	2.5th percentile	25th percentile	75th percentile	97.5th percentile
*Total population*							
Age (yrs.)	Total	2.07	1.84	0.65	1.34	2.57	4.58
	8–11	2.15	1.97	0.79	1.44	2.66	4.65
	12–14	2.20	2.01	0.65	1.44	2.79	4.62
	15–18	1.82	1.59	0.51	1.18	2.30	4.36

*Boys*							
Age (yrs.)	Total	2.14	1.95	0.71	1.42	2.62	4.90
	8–11	2.29	2.09	0.94	1.53	2.82	5.24
	12–14	2.14	1.99	0.53	1.43	2.66	4.81
	15–18	1.99	1.71	0.73	1.30	2.35	5.41

*Girls*							
Age (yrs.)	Total	1.98	1.75	0.62	1.28	2.47	4.55
	8–11	2.02	1.83	0.77	1.36	2.52	4.28
	12–14	2.27	2.11	0.89	1.45	2.81	4.57
	15–18	1.65	1.47	0.49	1.04	2.19	4.13

**Table 4:** Multivariate regression analysis with TSH as a dependent variable.

	Beta	Std error	*p* value
Total population			
Constant	2.44	0.18	<0.0001
BMI	0.004	0.001	0.001
Age	−0.06	0.011	<0.0001
SES	0.11	0.04	0.008
Sex	−0.16	0.07	0.018
Thyroid antibodies	0.17	0.16	0.28

Boys			
Constant	2.351	0.265	<0.0001
BMI	0.005	0.002	0.003
Age	−0.058	0.017	0.001
SES	0.128	0.057	0.026
Thyroid antibodies	−0.349	0.282	0.217

Girls			
Constant	2.399	0.248	<0.0001
BMI	0.002	0.002	0.145
Age	−0.059	0.015	<0.0001
SES	0.093	0.056	0.097
Thyroid antibodies	0.469	0.192	0.015
